# Supplementary material for: Polypyrimidine tract-binding proteins are essential for B cell development
Source: eLife. 2020 Feb 21;9:e53557. doi: 10.7554/eLife.53557 (PMC7058386; doi:10.7554/eLife.53557)
Supplement: Supplementary file 3. [file elife-53557-supp3.docx]

| **Key Resources Table** | | | | |
| --- | --- | --- | --- | --- |
| **Reagent type (species) or resource** | **Designation** | **Source or reference** | **Identifiers** | **Additional information** |
| antibody | Anti-PTBP1 (mouse IgG1, CLONE 1) | ThermoFisher Scientific | Cat# 32-4800 | FACS (1:400) |
| antibody | Anti-PTBP2 (mouse IgG2a, S43) | Michele Solimena |  | FACS (1:400, conc. 2.5 µg/ml) |
| antibody | Anti-PTBP3  (rat IgG2a, MAC454) | Martin Turner (ref. Monzón-Casanova, et al. Nat. Immunol. 45, 471 (2018). PMID: 29358707) |  | FACS (1:400 conc. 1.25 µg/ml) |
| other | Alexa Fluor™ 488 antibody labelling kit | ThermoFisher | Cat# A20181 |  |
| other | Alexa Fluor™ 647 antibody labelling kit | ThermoFisher | Cat# A20186 |  |
| antibody | B220-BUV395 (RA3-6B3, monoclonal, Rat IgG2a, κ) | BD Biosciences | Cat# 563793 | FACS (1:200) |
| antibody | CD19-BUV737 (1D3, monoclonal, IgG2a, κ) | BD Biosciences | Cat# 564296 | FACS (1:400) |
| antibody | CD43-biotin (S7, monoclonal, rat IgG_2a_, κ) | BD Biosciences | Cat# 553269 | FACS (1:200) |
| antibody | CD93-BV421 (AA4.1, monoclonal, rat IgG2b, κ) | BD Biosciences | Cat# 563806 | FACS (1:400) |
| antibody | IgM-PE Goat F(ab')₂ (Igu) (Polyclonal) | Jackson | Cat# 115-116-075 | FACS (1:400) |
| antibody | IgM-FITC Goat F(ab')₂ (Igu) (Polyclonal) | Jackson | Cat# 115-095-075 | FACS (1:400) |
| antibody | IgM-BV785 (R6-60.2, monoclonal, rat IgG2a, κ) | BD Biosciences | Cat# 564028 | FACS (1:50) |
| antibody | CD24-FITC (30F1, monoclonal, rat IgG2c, κ) | eBioscience | Cat# 11-0241-85 | FACS (1:50) |
| antibody | IgD-PerCP-Cy5.5 (11-26c.2a, monoclonal, Rat IgG2a, κ) | BioLegend | 405710 | FACS (1:400) |
| antibody | CD2-BV421 (RM2-5, monoclonal, rat IgG2b, λ) | BD Biosciences | 740010 | FACS (1:200_ |
| antibody | CD25-BV650 (PC61, monoclonal, Rat IgG1, λ) | BioLegend | 102038 | FACS (1:800) |
| antibody | BP-1-PE (BP-1, monoclonal, mouse IgG2a, κ) | BD Biosciences | 553735 | FACS (1:100) |
| antibody | Gr-1-AF594 (Ly-6G/Ly-6C, monoclonal, rat IgG2b, κ) | BioLegend | 108448 | FACS (1:200) |
| antibody | CD11b-AF594 (M1/70, monoclonal, rat IgG2b, κ) | BioLegend | 101254 | FACS (1:200) |
| antibody | Siglec-F-PE-CF594 (E50-2440, monoclonal, rat IgG2a, κ) | BD Biosciences | 562757 | FACS (1:400) |
| antibody | F4/80-AF594 (BM8, monoclonal, rat IgG2a, κ) | BioLegend | 123140 | FACS (1:400) |
| antibody | NK1.1-PE-Dazzle594 (PK136, monoclonal, mouse IgG2a, κ) | BioLegend | 108747 | FACS (1:400) |
| antibody | cKIT-APC (ACK2, monoclonal, rat IgG2b, κ) | BioLegend | 135108 | FACS (1:100) |
| antibody | IgD-PE (11-26, monoclonal, rat IgG2a, κ) | eBioscience | 12-5993-82 | FACS (1:400) |
| antibody | BrdU-AF647 (Mobu-1, monoclonal, mouse IgG) | ThermoFisher Scientific | B35133 | FACS (1:25) |
| antibody | CD19-PerCPCy5.5 (1D3, rat IgG2a, κ) | BioLegend | 152406 | FACS (1:400) |
| antibody | IgM-biotin (R6-60.2, monoclonal, rat IgG2a, κ) | BD Biosciences | 553406 | FACS (1:200) |
| antibody | CD25-biotin (PC61.5, monoclonal, rat IgG1, lambda) | eBioscience | 13-0251-85 | FACS (1:100) |
| antibody | Gr-1-biotin (RB6-8C5, monoclonal, rat IgG2b, κ) | eBioscience | 13-5931 | FACS (1:800) |
| antibody | IgD-biotin (11-26c.2a, monoclonal, rat Rat IgG2a, κ) | Southern Biotech | 1120-08 | FACS (1:400) |
| antibody | CD2-biotin (RM2-5, monoclonal, rat IgG2b, λ ) | BioLegend | 100104 | FACS (1:400) |
| antibody | CD11b-biotin (M1/70, monoclonal, rat IgG2b, k) | eBioscience | 13-0112-82 | Depletion (1:400) |
| antibody | NK1.1-biotin (PK136, monoclonal, mouse IgG2a, κ) | BD Biosciences | 553163 | Depletion (1:400) |
| antibody | CD3e-biotin (145-2C11, monoclonal, hamster IgG1, κ) | BD Biosciences | 553060 | Depletion (1:200) |
| antibody | Ter119-biotin (monoclonal, rat IgG2b, k) | eBioscience | 13-5921-82 | Depletion (1:100) |
| antibody | Phospho-Histone H3 (Ser10) (Rabbit polyclonal) | Cell Signalling | 9701S | FACS (1:50) |
| antibody | Donkey anti-Rabbit IgG (H+L)-AF647 (Polyclonal) | Jackson | 711-606-152 | FACS (1:400) |
| antibody | pH2A.X-phosphorylated-AF647 (2F3, monoclonal, mouse IgG1, κ) | BioLegend | 613408 | FACS (1:10) |
| antibody | mouse IgG1-AF647 Isotype control (monoclonal) | BioLegend | 400155 | FACS (diluted as primary to control for) |
| antibody | p53-AF647 (1C12, monoclonal, mouse Mouse IgG1) | Cell Signaling Technology | 2533S | FACS (1:50) |
| antibody | Rabbit (DA1E) mAb IgG XP® -AF647 (Rabbit monoclonal IgG) | Cell Signaling Technology | 2985S | FACS (diluted as primary to control for) |
| antibody | cMYC-AF647 (D84C12, monoclonal, rabbit IgG) | Cell Signaling Technology | 13871S | FACS (1:50) |
| antibody | CD43-BV421 (S7, monoclonal, rat IgG2a, κ) | BD Biosciences | 562958 | FACS (1:50) |
| antibody | c-KIT-BV510 (ACK2, monoclonal IgG2b, κ) | BioLegend | 135119 | FACS (1:50) |
| antibody | CD25-BV785 (PC61, monoclonal, Rat IgG1, λ) | BD Biosciences | 564023 | FACS (1:50) |
| antibody | IgM-PECy7 (II/41, monoclonal rat IgG2a, κ) | eBioscience | 25-5790-81 | FACS (1:200) |
| antibody | CD2-PECy7 (RM2, monoclonal rat IgG2b, λ) | BioLegend | 100113 | FACS (1:200) |
| antibody | CD127 (IL7R)-PECy7 (A7R34, monoclonal rat IgG2a, κ) | BioLegend | 135014 | FACS (1:200) |
| antibody | B220-APC (RA3-6B2, monoclonal, Rat IgG2a, κ) | Tonbo | 20-0452-U100 | FACS (1:200) |
| antibody | p27 (KIP1) (Y236, rabbit monoclonal) | Abcamb | ab32034 | FACS (1:200) |
| antibody | p-SAMHD1 (Thr592) (D7O2M,monoclonal, rabbit IgG ) | Cell Signaling Technology | 89930S | FACS (1:800) |
| antibody | p-Rb (Ser807/811)-AF647 (D20B12) XP® (monoclonal, rabbit IgG) | Cell Signaling Technology | 8974S | FACS (1:50) |
| other | Streptavidin-PECy7 | BD Biosciences | 557598 | FACS (1:400) |
| other | Streptavidin-BV510 | BioLegend | 405234 | FACS (1:400) |
| other | Live/Dead staining (eFluor780) | eBioscience | 65-0865-18 | FACS (1:2000) |
